# Correction: The local burden of disease during the first wave of the COVID-19 epidemic in England: estimation using different data sources from changing surveillance practices

**DOI:** 10.1186/s12889-022-13320-8

**Published:** 2022-06-07

**Authors:** Emily S. Nightingale, Sam Abbott, Timothy W. Russell, CMMID Covid-19 Working Group, Eleanor M. Rees, Rosalind M. Eggo, Matthew Quaife, Fiona Yueqian Sun, Carl A. B. Pearson, Kiesha Prem, James D. Munday, Sophie R. Meakin, Kevin van Zandvoort, W. John Edmunds, Alicia Rosello, Sebastian Funk, Kathleen O’Reilly, Billy J. Quilty, Simon R. Procter, Amy Gimma, Adam J. Kucharski, Arminder K. Deol, Jon C. Emery, Nikos I. Bosse, Hamish P. Gibbs, David Simons, Stéphane Hué, Christopher I. Jarvis, Petra Klepac, Yang Liu, Anna M. Foss, Charlie Diamond, C. Julian Villabona-Arenas, Akira Endo, Rein M. G. J. Houben, Stefan Flasche, Samuel Clifford, Gwenan M. Knight, Joel Hellewell, Nicholas G. Davies, Katherine E. Atkins, Damien C. Tully, Megan Auzenbergs, Mark Jit, Rachel Lowe, Graham F. Medley, Oliver J. Brady

**Affiliations:** 1 Department of Global Health and Development, London School of Hygiene and Tropical Medicine, London, UK; 2 Centre for Mathematical Modelling of Infectious Disease (CMMID), London School of Hygiene & Tropical Medicine, London, UK; 3 Department of Infectious Disease Epidemiology, London School of Hygiene and Tropical Medicine, London, UK; 4 Centre on Climate Change and Planetary Health, London School of Hygiene & Tropical Medicine, London, UK; 5 Barcelona Supercomputing Centre (BSC), Barcelona, Spain; 6 Catalan Institution for Research and Advanced Studies (ICREA), Barcelona, Spain

**Correction to: BMC Public Health 22, 716 (2022)**

**https://doi.org/10.1186/s12889-022-13069-0**

The original publication of this article [1] contained 2 errors:

1. 3 main authors were shown as collaborators, this affected Rachel Lowe, Graham F. Medley and Oliver J. Brady
2. The full collaborator list was not available

The original article has been updated with the correct information.
